# Supplementary material for: Distinct Changes in Placental Ceramide Metabolism Characterize Type 1 and 2 Diabetic Pregnancies with Fetal Macrosomia or Preeclampsia
Source: Biomedicines. 2023 Mar 17;11(3):932. doi: 10.3390/biomedicines11030932 (PMC10046505; doi:10.3390/biomedicines11030932)
Supplement: Supplementary file 1 [file biomedicines-11-00932-s001.zip › Supplementary Figure S1.pdf]

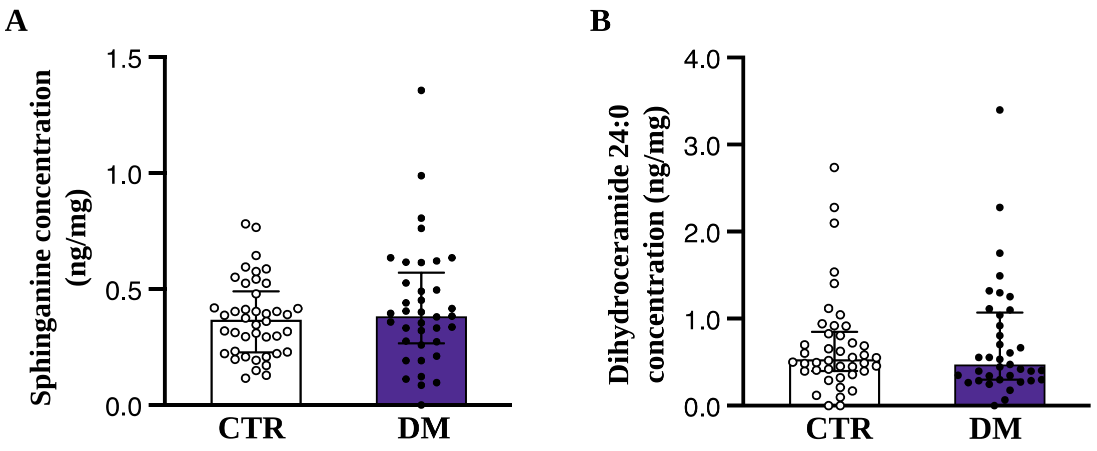

Figure S1. Intermediates of the *de novo* pathway of ceramide synthesis. (**A**) sphinganine and (**B**) dihydroceramide 24:0 concentrations in control *vs.* diabetic (type 1 and 2) placentae.
