# Supplementary material for: Distinct Changes in Placental Ceramide Metabolism Characterize Type 1 and 2 Diabetic Pregnancies with Fetal Macrosomia or Preeclampsia
Source: Biomedicines. 2023 Mar 17;11(3):932. doi: 10.3390/biomedicines11030932 (PMC10046505; doi:10.3390/biomedicines11030932)
Supplement: Supplementary file 1 [file biomedicines-11-00932-s001.zip › Supplementary Figure S2.pdf]

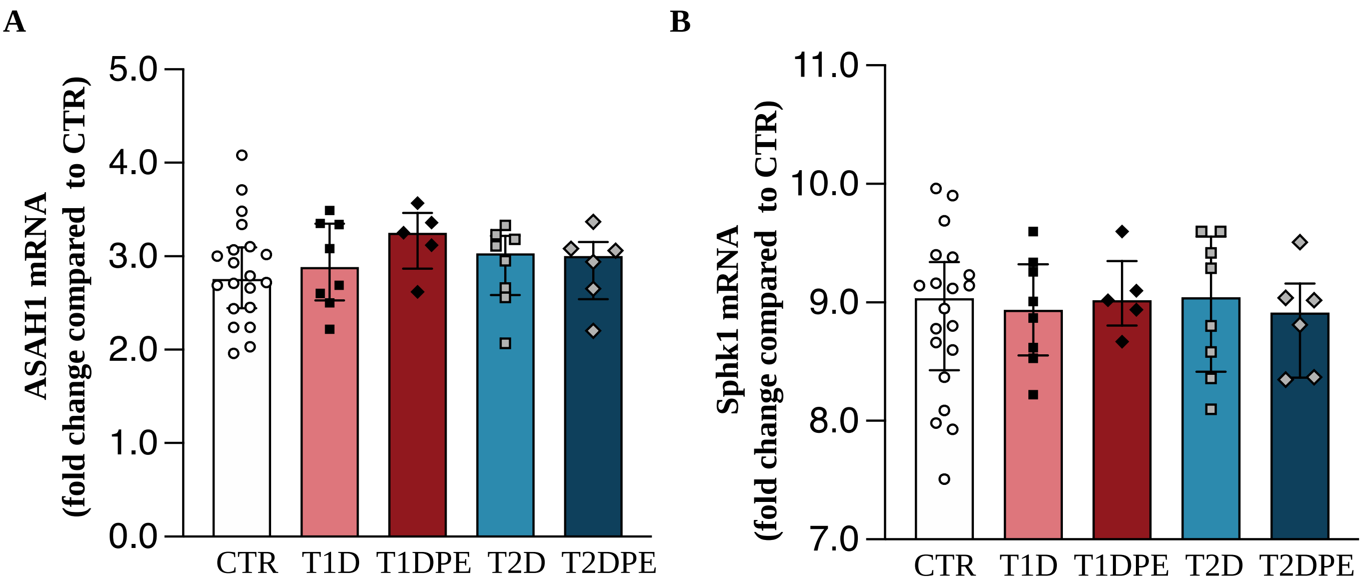

Figure S2. ASAH1 and SPHK mRNA expression in type 1 and type 2 diabetic placentae. qPCR analysis of (**A**) placental acid ceramidase (ASAH1) and (**B**) sphingosine kinase 1 (SPHK1) mRNA levels.
