# Supplementary material for: Distinct Changes in Placental Ceramide Metabolism Characterize Type 1 and 2 Diabetic Pregnancies with Fetal Macrosomia or Preeclampsia
Source: Biomedicines. 2023 Mar 17;11(3):932. doi: 10.3390/biomedicines11030932 (PMC10046505; doi:10.3390/biomedicines11030932)
Supplement: Supplementary file 1 [file biomedicines-11-00932-s001.zip › Supplementary Table S1.pdf]

Table S1. Placental concentrations of ceramide and bioactive lysosphingolipids in obese pregnancies compared to lean CTR pregnancies.

**Ceramides**	**Non-obese CTR** (n=27)	**Obese CTR** (*n*=7)	*p* value
Total CER	55.32 (42.66/67.03)	50.18 (34.83/63.84)	0.901
CER 14:0	0.12 (0.08/0.16)	0.11 (0.09/0.14)	0.677
CER 16:0	7.58 (5.62/9.24)	6.70 (5.38/8.47)	0.901
CER 18:0	1.07 (0.76/1.46)	1.52 (1.00/1.84)	0.201
CER 20:0	0.59 (0.51/0.91)	0.59 (0.47/1.19)	0.901
CER 22:0	9.24 (6.20/14.50)	7.54 (6.04/12.83)	1.000
CER 24:0	23.40 (16.67/28.30)	20.20 (14.26/23.90)	0.452
CER 24:1	12.60 (8.99/14.65)	11.62 (9.62/15.75)	0.771
**Sphingoid-1-phosphates**	**Non-obese CTR** (n=11)	**Obese CTR** (*n*=6)	*p* value
S1P+Sa1P	0.086 (0.055/0.089)	0.097 (0.067/0.149)	0.301

Lipid concentrations (ng/mg of tissue). Values are median (interquartile range). Obese group: BMI 32.1-64.1 kg/m^2; lean group: BMI 17.2-27.7 kg/m^2. Ceramides (CER); Total sphingoid-1-phosphates (Total sphingoid-1-P); Sphingosine-1-phosphate (S1P); sphinganine-1-phosphate (Sa1P).
